# Comparison of Testing Designs for Flexural Strength of 3Y-TZP and 5Y-PSZ Considering Different Surface Treatment

**DOI:** 10.3390/ma15113915

**Published:** 2022-05-31

**Authors:** Carsten Hergeröder, Sebastian Wille, Matthias Kern

**Affiliations:** Department of Prosthodontics, Propaedeutics and Dental Materials, School of Dentistry, Christian-Albrechts University, 24105 Kiel, Germany; swille@proth.uni-kiel.de (S.W.); mkern@proth.uni-kiel.de (M.K.)

**Keywords:** zirconia, 3Y-TZP, 5Y-PSZ, flexural strength testing

## Abstract

The aim of this study was to analyze the influence of different surface treatments and the corresponding surface roughness on the ball-on-three-balls test and piston-on-three-balls test for measuring flexural strength 3Y-TZP and 5Y-PSZ. Additionally, the influence of cutting the material into the specimens when pre-sintered or fully sintered was analyzed. A total of 120 specimens for each material group, 3Y-TZP zirconia (Katana HT, Kuraray) and the 4 different layers of multilayered 5Y-PSZ zirconia (Katana UTML, Kuraray), were produced. The used material was cut into half of the specimens in a fully sintered stage, the other half was cut when pre-sintered. Each subgroup was divided into 3 different surface treatment groups being air abraded with 50 µm alumina particles at 1 bar pressure, ground with 600 SiC paper or polished up to 1 µm with a polycristalline diamond suspension. These were then analyzed by X-ray diffraction (XRD) (N = 3) and tested for flexural strength using the ball-on-three-balls test (N = 10) or piston-on-three-balls test (N = 10). For 3Y-TZP groups different surface roughness did not result in statistically significant differences in flexural strength but cutting the specimens in fully sintered state significantly reduced flexural strength of 1133 ± 109 to 741 ± 81 MPa. For 5Y-PSZ groups air abrasion of the specimens with alumina particles significantly reduced the flexural strength of 562 ± 68 MPa to 358 ± 58 MPa. Cutting and surface treatment in pre-sintered or fully sintered state had no significant influence. Flexural strength testing with the ball-on-three-balls test resulted in about 20% higher strengths compared to the piston-on-three-balls test. Results of both tests showed the same tendencies when compared.

## 1. Introduction

In dentistry, the demand for esthetic and natural looking materials is increasing. For this reason, tooth-colored restorations are receiving more interest in comparison to metal and metal-ceramic restorations. Ceramics are therefore becoming increasingly popular. Oxide ceramics have high strength, especially yttrium-stabilized tetragonal zirconia polycrystalline ceramic (Y-TZP). The advantage of adding yttrium oxide is that it stabilizes the ceramic against damage as it occurs. When mechanical or thermal impact occurs, the microstructure changes from tetragonal to monoclinic in the region of the ongoing fracture. This leads to a local compressive stress, then to a counterforce and a fracture stop [1,2].

Compared to enamel and dentin, zirconia is more opaque [3]. To improve translucency, a higher amount (5 mol%) of yttrium oxide is added, making it 5 yttria stabilized zirconia (5Y-PSZ). This results in better translucency, but also reduced flexural strength compared to 3Y-TZP. The reduced amount of tetragonal phase reduces the fracture toughness [4].

For 3Y-TZP, many studies investigated a possible influence on the material when it is treated pre-sintered or fully sintered [5,6,7]. According to these studies the treatment of fully sintered 3Y-TZP, for example, air-abrasion, leads to reduced flexural strength.

An important parameter for the evaluation of dental zirconia is the flexural strength. Various methods have been established for analyzing flexural strength, for example, the three-point bending test and four-point bending test as uniaxial tests [8,9,10]. However, the piston-on-three-balls test is typically used to measure the biaxial flexural strength [5,11,12,13]. Biaxial tests have prevailed over uniaxial tests because of their greater tolerance in specimen preparation and the easier miniaturization [14]. The possible anisotropy of a specimen would not be measured in a uniaxial test. For flaws in the specimen that are not in the tested loading axis, this would not result in a lower flexural strength, grinding of 3Y-TZP specimens reduced their flexural strength [14,15,16].

The influence of different surface treatments of zirconia on the flexural strength was analyzed [17]. The specimens were polished or treated with air-abrasion. Polished zirconia specimens showed higher flexural strength than those air-abraded with 50 μm alumina particles. In the above studies, the surface roughness of the specimens was evaluated for a possible influence on the flexural strength. However, a possible influence of surface roughness on the flexural strength testing design was not evaluated.

A comparison of flexural strength testing setups showed a higher flexural strength for 3Y-TZP with the ball-on-three-balls test instead of the piston-on-three-balls test [18,19]. A possible reason for this result could be the influence of surface roughness on the piston-on-three-balls test, which was tested with a specific surface roughness. An objective of this study was to evaluate a possible influence of surface roughness of 3Y-TZP and 5Y-PSZ on the piston-on-three-balls test and ball-on-three-balls testing setup.

For 5Y-PSZ the flexural strength was only investigated after hydrothermal aging or different sintering procedures [20,21,22].

Since the treatment of 5Y-PSZ in the fully sintered or pre-sintered condition in the aspect of flexural strength has not been analyzed yet, another objective of this study was to investigate a possible influence of treatment in different sintering stages on flexural strength.

Concluding from this, the null hypothesis is that there is no difference in flexural strength between the two test setups and that there is no influence of the different surface treatment on the test setup.

## 2. Materials and Methods

### 2.1. Specimen Fabrication

In this study, Katana HT was used as 3Y-TZP and Katana UTML (both Kuraray, Tokyo, Japan) was used as 5Y-PSZ. Since 5Y-PSZ was a multicolor material, the factory disks were cut into the different color layers and tested separately. The different LOT Numbers and subgroups are listed in Table 1.

**Figure 1:**
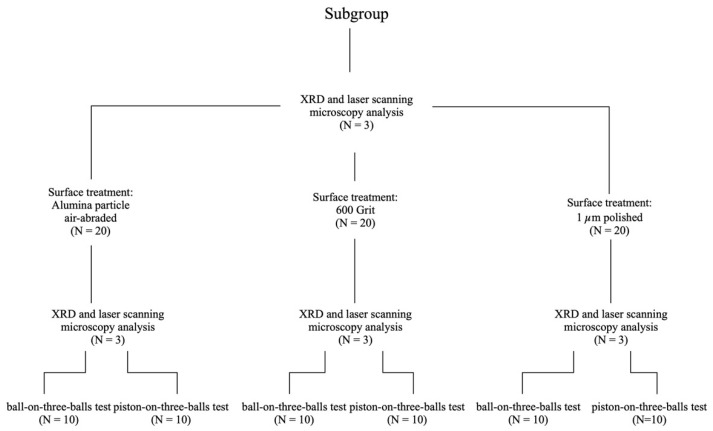
Flowchart of the specimen groups. Subgroups being 3Y-TZP sintered before and after cutting and the different Layers of 5Y-PSZ sintered before and after cutting.

For each material 120 specimens were fabricated. To analyze the influence of cutting the specimens at different sintering stages on the flexural strength, half of the specimens were fully sintered before cutting, the other half were fully sintered after cutting. The specimens were milled with a final diameter of 12 mm when fully sintered from all four layers of the multilayered 5Y-PSZ and of the 3Y-TZP single layered material. For the milling process, an automated milling machine (Zenotec Hydro, Wieland Dental, Pforzheim, Germany) was used. The four 5Y-PSZ layers consisted of enamel layer, transition layer 1 (TL1), transition layer 2 (TL2) and dentin layer. Since the material was milled from the factory disks into cylinders with a thickness of 22 mm, they had to be cut into specimens of nearly 1.2 mm thickness in the fully sintered state using the IsoMet Speed Pro (Buehler, IL, USA). The specimens were divided into two groups: for one group, the cylinders were cut to the specimen thickness before the final sintering process, and the other group was cut after being fully sintered. The sintering process followed the manufacturer’s instructions (1500 °C for 3Y-TZP, 1550 °C for 5Y-PSZ; holding time 2 h). The specimens were then leveled to a thickness of 1.2 ± 0.02 mm using the EcoMet 250 Pro (Buehler, IL, USA). The specimens of the different layers were then divided into 3 different subgroups (N = 20) for surface treatment. Specimens were treated on both sides. One group was treated with SiC Paper 600 Grit with water cooling using the EcoMet 250 Pro (Buehler, IL, USA); 1 group was polished up to 1 µm with polycrystalline diamond suspension MetaDi Supreme (Buehler, IL, USA). The last group was treated with air abrasion with alumina particles of 50 µm at a pressure of 1 bar from a distance of 10 mm. For this purpose, the specimens were marked with a dye pen and then air abraded until the color fully disappeared. A flowchart of the different groups and subgroups is shown in Figure 1.

### 2.2. Surface Analysis

To analyze the surface roughness obtained, 3 specimens from each group were randomly selected and examined using a laser scanning microscope (VK-X100, Keyence, Osaka, Japan) at a laser wavelength of 658 nm. The mean R_a_ value of the three specimens of each group was evaluated. In addition, possible color variation between the specimens was visually analyzed if they were cut before or after final sintering.

### 2.3. Analysis of Grain Size

To acknowledge a possible change in grain size in the different layers of the multilayered ceramic images with a scanning electron microscope Zeiss Ultra Plus (Carl Zeiss AG, Oberkochen, Germany). The measurement was performed with an acceleration voltage of 5 kV with a measuring distance of 7.5 mm. Three specimens of each layer were analyzed.

### 2.4. Structural Analysis

To evaluate possible phase changes due to surface treatment, the specimens were subjected to X-ray diffraction. For this purpose, three randomly selected specimens from each group were measured before and again after surface treatment using the Smartlab (Rigaku, Tokyo, Japan) to investigate the crystalline structure. The measurement range for 2θ was 20° to 40° in steps of 0.04°.

### 2.5. Flexural Strength Testing

After surface treatment the specimens were divided randomly into 2 subgroups each (N = 10) and tested for flexural strength in two different flexural strength tests. A universal testing machine (Z010, Zwick, Ulm, Germany) with a speed of 0.5 mm/min was used to measure the force in both tests.

### 2.6. Piston-on-Three-Balls Test

For the piston-on-three-balls test the specimens were positioned on 3 balls with the radius r = 3.4 mm and a resulting loading circle with a diameter of 10 mm. The force was applied by a flat piston with a diameter of 1.5 mm. The flexural strength (in MPa) was calculated using the equation [23]:

(1) $$\sigma \;=-0.2387\times P\frac{X-Y}{b^{2}}$$

Parameters were: P: Loading force measured in the experimental setup, b: thickness of the specimen (mm), *X* = (1 + v)·ln(r_2_/r_3_)^2^ + [(1 − v)/2]·(r_2_/r_3_)^2^, *Y* = (1 + v)·[1 + ln(r_1_/r_3_)^2^] + (1 − v)·(r_1_/r_3_)^2^, v: Poisson ratio (0.25), r_1_: radius of the loading circle (mm), r_2_: radius of the applied force area (mm), r_3_: radius of the specimen (mm).

### 2.7. Ball-on-Three-Balls Test

For the other subgroup (N = 10), the biaxial flexural strength was measured with the ball-on-three-balls test. For this purpose, the specimens were positioned on 3 equally sized balls with a diameter of 8 mm. The force was applied by another ball of the same size with a diameter of 8 mm. The results were then evaluated using the tool provided by the Montan University [14].

### 2.8. Statistical Analysis

Different methods for the statistical evaluation were used for the results of the flexural strength tests. The Shapiro-Wilk Test was used to test the results for normal distribution and with the Levene Test for homogeneity of variances. For the statistical level *p* ≤ 0.05 was used. The results showed no normal distribution for only a few groups and no homogenous distribution between groups. Therefore, the results were analyzed with one-way ANOVA and Games-Howell post-hoc analysis.

## 3. Results

### 3.1. Sintering State during Cutting Process

**Figure 2:**
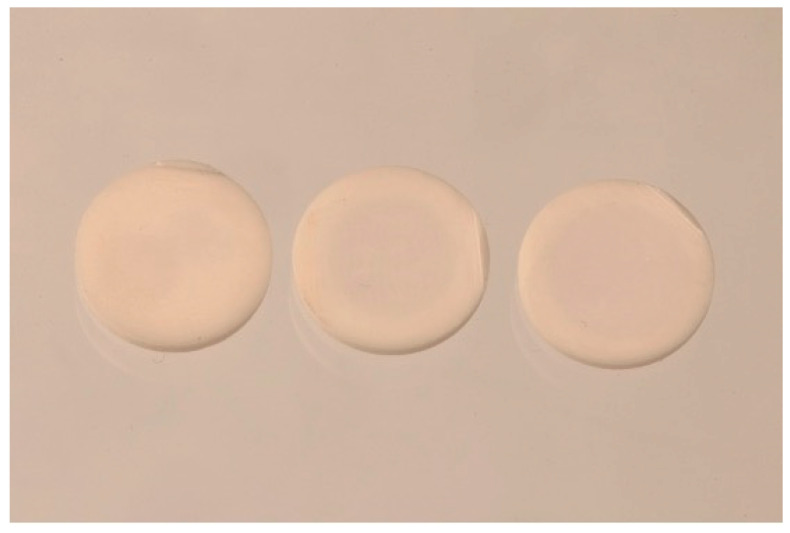
Photo of three specimens being sintered before the specimens are cut to the final thickness. The specimens are of different depth of the finally sintered cylinder of 3Y-TZP with the same diameter and thickness. Different color saturation can be seen from right to left.

The cutting process of pre-sintered or fully sintered specimens showed a significant difference only for 3Y-TZP, not for 5Y-PSZ. For 3Y-TZP cutting the specimens when pre-sintered resulted in a higher flexural strength. Cutting fully sintered specimens also resulted in an inhomogeneous color saturation of the specimens as shown in Figure 2.

### 3.2. Surface Roughness Evaluation

The analysis of surface roughness with the laser scanning microscope showed detectable variations as shown in Table 2. Similar surface roughness of 3Y-TZP and 5Y-PSZ groups was measured. Only after grinding the specimens with 600 SiC paper was the R_a_ value for 3Y-TZP was lower in comparison to 5Y-PSZ.

### 3.3. Analysis of Grain Size

In Table 3 the mean grain sizes for the different layers of 5Y-PSZ and for 3Y-TZP are listed. The results show no significant difference in between the layers of 5Y-PSZ.

### 3.4. Phase Analysis

**Figure 3:**
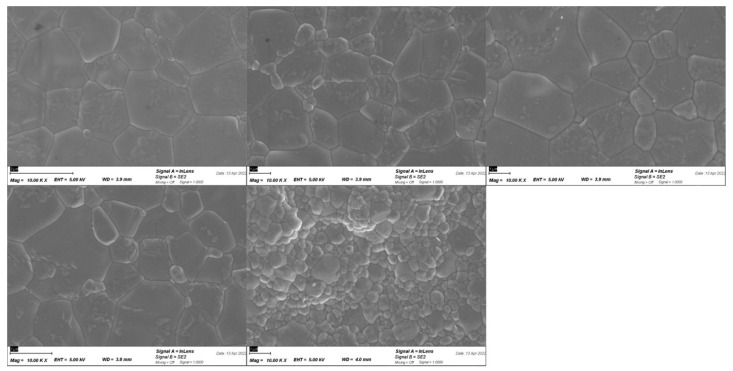
SEM Image of the different layers of 5Y-PSZ and 3Y-TZP.

**Figure 4:**
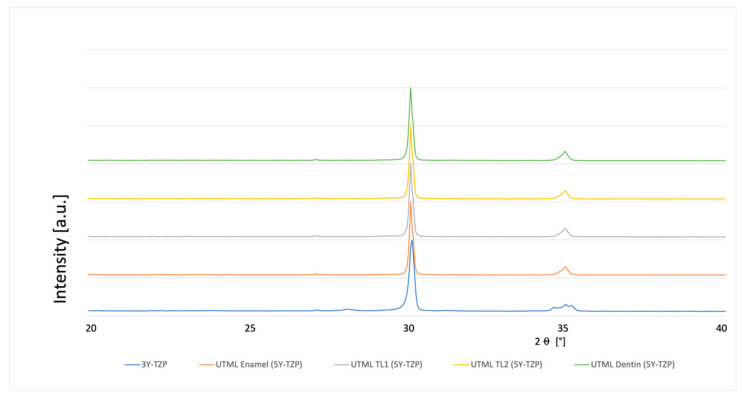
XRD analysis 3Y-TZP and 5Y-PSZ 1 µm polished and fully sintered before cutting.

**Figure 5:**
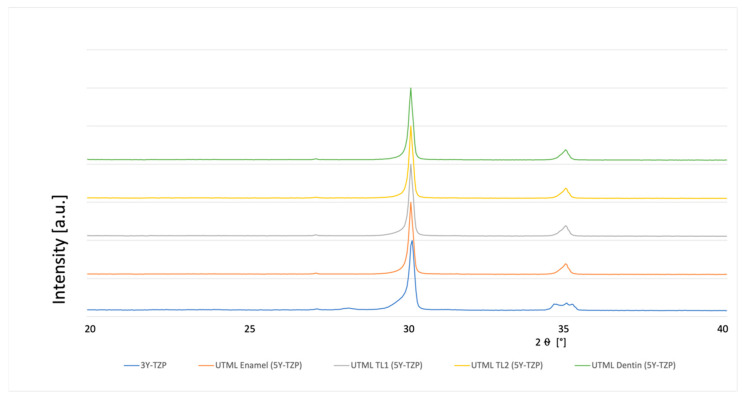
XRD analysis 3Y-TZP and 5Y-PSZ 600 Grit and fully sintered before cutting.

**Figure 6:**
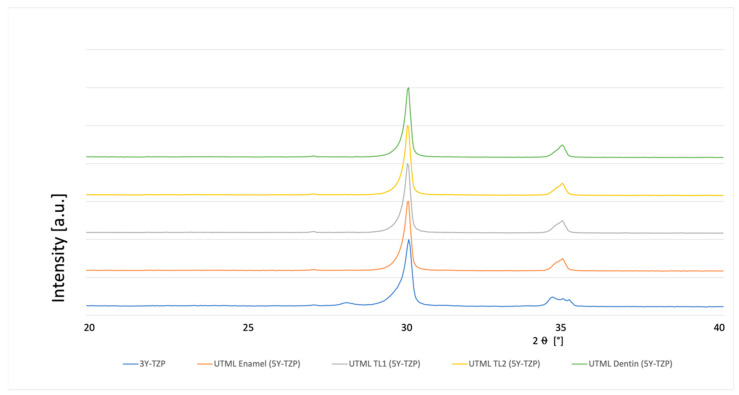
XRD analysis 3Y-TZP and 5Y-PSZ air abraded with alumina particles 50 µm and fully sintered before cutting.

**Figure 7:**
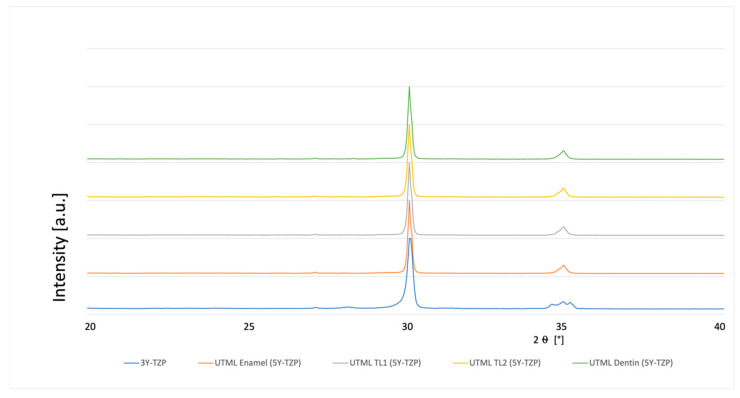
XRD analysis 3Y-TZP and 5Y-PSZ polished up to 1 µm and pre-sintered at cutting.

**Figure 8:**
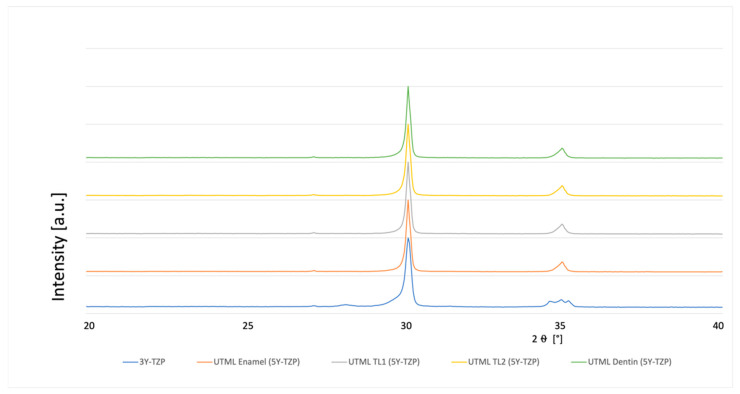
XRD analysis 3Y-TZP and 5Y-PSZ 600 Grit and pre-sintered at cutting.

**Figure 9:**
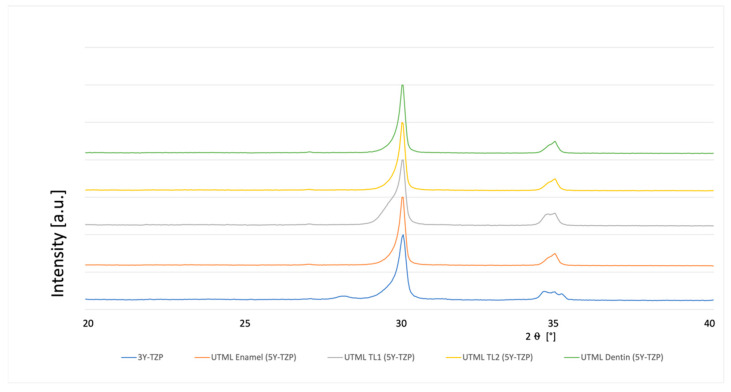
XRD analysis 3Y-TZP and 5Y-PSZ air abraded with alumina particles 50 µm and pre-sintered at cutting.

A typical XRD spectrum for the different materials is shown in Figure 3, Figure 4, Figure 5, Figure 6, Figure 7, Figure 8 and Figure 9. The XRD analysis showed no significant difference between the groups, neither for the pre-sintered or fully sintered, nor after different surface treatment. Based on the existing peaks compared with current studies, specimens from 5Y-PSZ showed a cubic phase whereas 3Y-TZP specimens showed a tetragonal structure. Only minimal phase changes for the 3Y-TZP specimens were detected [21,24].

### 3.5. Biaxial Flexural Strength Testing

Table 4 and Table 5 show the mean values of the flexural strength of the test groups measured by the piston-on-three-balls test or the ball-on-three-balls test, respectively.

The different surface treatments only showed a statistically significant difference for 5Y-PSZ between specimens air-abraded with alumina particles and polished to 1 µm or ground with 600 SiC paper. Surface roughness had no influence on biaxial flexural strength in the other subgroups.

The ball-on-three-balls test showed about 20% higher flexural strength than the piston-on-three balls test. The results of both tests showed the same tendency for all test groups and the differences occurred in both testing setups.

## 4. Discussion

The null hypothesis must be rejected since the two testing setups led to different flexural strength of the materials. Furthermore, alumina particle air-abrasion significantly reduced the flexural strength of 5Y-PSZ.

Conventional 3Y-TZP was used as the control group. The flexural strength for the 3Y-TZP is affirmative with multiple studies, which ensures the reliability of the test method [5,13]. For the 3Y-TZP in each different group the treatment of cutting the specimens pre-sintered or fully sintered made a statistically significant difference on the flexural strength. Cutting the fully sintered 3Y-TZP reduced the flexural strength, confirming results published by Aboushelib and Wang [9]. In their study, the flexural strength of zirconia was tested after grinding with a diamond point, which is comparable to the cutting of fully sintered 3Y-TZP performed in this study. For fully sintered 3Y-TZP, the force applied for cutting was higher because of the higher strength of the material. This results in higher stress for the material leading to reduced flexural strength. Microcracks caused by the treatment of 3Y-TZP in the pre-sintered stage are subsequently closed during the final sintering process. The crack propagation could not be stopped by phase transformation observed after surface treatment, possibly due to the high force and the lack of healing of the microcracks when the specimens are already finally sintered [13]. In an overview on strength of ceramics possible reasons for a decrease in flexural strength are described. As described in the overview, the machining can lead to defects of specimens and thus to a reduced flexural strength [25]. Another possible reason could be an induced higher internal stress of the specimen during treatment in the fully sintered state, which was not investigated in this study.

The flexural strength results for 5Y-PSZ show the similar values when the specimens were not alumina particle air-abraded compared to existing studies [26,27]. This confirms the correct production of the specimen and the course of the test.

The SEM analysis showed a significantly different grain size for 3Y-TZP in comparison to 5Y-PSZ. The difference in grain size is consistent with previous studies [28,29].

Monolithic zirconia is usually milled and treated in the pre-sintered state. In the experimental setup each different layer of the 5Y-PSZ did not show any significant difference whether they were treated pre-sintered or fully sintered, no matter which surface treatment was applied. A possible reason for this could be the cubic phase structure in comparison to the tetragonal phase of the 3Y-TZP [30]. Since the force applied to cut the specimens of 5Y-PSZ is lower compared to 3Y-TZP, this might have led to a reduced damage and fewer microcracks in the material. The specimens which were treated in a fully sintered state showed color degradation towards the center of the specimen. This could be due to a lack of oxygen diffusion towards the center during the sintering process. However, the flexural strength showed no significant difference for the 5Y-PSZ being treated in a pre-sintered or fully sintered state. Dental restorations should therefore be milled, cut in a pre-sintered state to achieve lower material consumption, higher flexural strength and optimal translucency and color especially for 3Y-TZP, as confirmed by this study.

A study of Karakoca et al. investigated different surface treatments of 3Y-TZP [13]. The specimens were treated with air-abrasion using alumina particles, ground with a diamant bur or had no treatment. The specimens were treated at 4 bar pressure in comparison to 1 bar, used in the current study. The results show an increase in flexural strength when treated with air abrasion with aluminum oxide particles. The R_a_ obtained was significantly higher in the study by Karakoca et al. [13]. A possible reason therefore could be the 4 times higher air pressure used for air abrasion, which might also lead to a higher extend of phase transformation. This could have led to a toughening of the material, compared to the present study, where no significant phase transformation occurred.

These results contrast with the study by Inokoshi et al. [31]. where the surface treatment led to a phase transformation towards a monoclinic phase. Possible reasons for the different results could be the use of different materials. Even in the study by Inokoshi et al., the various materials led to differing levels of phase transformation.

Biaxial flexural strength was significantly lower for the 5Y-PSZ specimens in each layer when treated with alumina particle air-abrasion compared to polishing and grinding. Since this difference occurred regardless of the layer and regardless of sintering before or after cutting in 5Y-PSZ but not for the 3Y-TZP specimens, these results should not be considered as an effect of surface roughness on the experimental setup but on the material itself. This could be due to irreparable damage from air-abrasion and the lack of phase transformation strengthening. The reduced flexural strength of 5Y-PSZ after air-abrasion should be critically considered for clinical usage.

A study of Börger et al. investigated the influence of various parameters on the ball-on-three-balls-test, for example, the friction forces between the specimen and the balls of the test setup [32]. It was found that the friction between the specimen and balls had only a minor influence on the measured flexural strength. Since the ball-on-three-balls test gave even higher results for the flexural strength, it seems that the influence of friction between specimen and the balls can be neglected.

In a study by Amarante et al., the effect of surface roughness on flexural strength was analyzed. The authors concluded that alumina particle air-abrasion reduced the flexural strength for 5Y-PSZ by 37.5% in comparison to polishing the specimens [26]. The study by Amarante et al. underlines the results of the current study, although alumina particles of different size were used, and the air pressure was three times higher. This could also be the reason for the differences in Ra measured in the studies.

The flexural strength for 3Y-TZP and 5Y-PSZ measured with the ball-on-three-balls test was significantly higher than the results obtained with the piston-on-three-balls test. This is due to a smaller effective volume when using the ball-on-three-balls test. This has already been described for the comparison between 4-point, 3-point, biaxial flexural strength tests [33].

Since there are no studies available to date on testing flexural strength with the B3B test, it is difficult to compare the obtained results. Further studies on the comparison and reliability of the tests are recommended. Accordingly, when specifying the biaxial flexural strength for a material, it should additionally be stated which test method was used similar to the different uniaxial flexural strength tests [18].

## 5. Conclusions

The following conclusions can be drawn:

For 3Y-TZP and 5Y-PSZ, different surface roughness had no influence on the experimental results for flexural strength for the piston-on-three-balls test and ball-on-three-balls test.

Piston-on-three-balls test and ball-on-three-balls test showed the same relations between different investigated groups for flexural strength, but about 20% higher results with the ball-on-three-balls test.

Alumina particle air-abrasion significantly reduced the flexural strength of 5Y-PSZ zirconia. Air-abrasion of 5Y-PSZ should therefore be caried out with caution.

Cutting pre-sintered or fully sintered 3Y-TZP zirconia had a significant influence on flexural strength. Therefore, 3Y-TZP zirconia should only be treated in the pre-sintered stage to achieve maximum flexural strength.

## Figures and Tables

**Table 1:** LOT numbers of the used materials.

Material	Type	LOT Number	ZrO_2_ (Wt%)	Y_2_O_3_ (wt%)
KATANA UTML fully-sintered	5Y-PSZ	DRJXC	87–92	8–11
KATANA HT fully-sintered	3Y-PSZ	DESUEX	90–95	5–8
KATANA UTML presintered	5Y-PSZ	DSUEX	87–92	8–11
KATANA HT presintered	3Y-TZP	DTGUO	90–95	5–8

**Table 2:** Means (SD) in µm (Ra) of Surface roughness measured with laser scanning microscopy.

Treatment	Sintering Stage at Treatment	3Y-TZP	5Y-PSZ Enamel	5Y-PSZ TL1	5Y-PSZ TL2	5Y-PSZ Dentin
1 µm polished	Fully sintered	0.025	0.025	0.018	0.024	0.025
		(0.012)	(0.006)	(0.000)	(0.004)	(0.003)
	Pre-sintered	0.023	0.014	0.013	0.020	0.028
		(0.007)	(0.001)	(0.005)	(0.003)	(0.011)
600 Grit SiC Paper grinded	Fully sintered	0.076	0.150	0.136	0.151	0.160
		(0.015)	(0.013)	(0.008)	(0.005)	(0.005)
	Pre-sintered	0.075	0.147	0.114	0.115	0.110
		(0.005)	(0.103)	(0.026)	(0.002)	(0.016)
Alumina particle air-abraded	Fully sintered	0.300	0.386	0.395	0.366	0.373
		(0.059)	(0.028)	(0.052)	(0.023)	(0.031)
	Pre-sintered	0.291	0.311	0.341	0.291	0.348
		(0.027)	(0.008)	(0.024)	(0.054)	(0.029)

**Table 3:** Grain size of the four layers of 5Y-PSZ and of 3Y-TZP in µm^2^.

Material	Enamel Layer (5Y-PSZ)	Transition Layer 1 (5Y-PSZ)	Transition Layer 2 (5Y-PSZ)	Dentin Layer (5Y-PSZ)	3Y-TZP
Mean (SD)	3.82	3.47	3.66	3.40	0.18
	(0.65)	(0.25)	(0.44)	(0.30)	(0.011)

**Table 4:** Medians, means and standard deviations (SD) in MPa of the biaxial flexural strength of test groups (N = 10) measured with piston-on-three-balls test. Statistically different medians (*p* ≤ 0.05) are indicated by different upper-case letters (within a column for analysis of the different materials with the same surface treatment), or by subscript lower-case letters (within a row for the same material with different surface treatment), or by different lower-case Greece letters (comparison of the cutting before and after sintering).

Treatment	Sintering Stage at Treatment	3Y-TZP		5Y-PSZ Enamel		5Y-PSZ TL1		5Y-PSZ TL2		5Y-PSZ Dentin	
		Median	Mean (SD)	Median	Mean (SD)	Median	Mean (SD)	Median	Mean (SD)	Median	Mean (SD)
1 µm polished	Fully sintered	719	718	470	474	514	494	500	486	494	481
		A, a, β	(40)	B, a, α	(39)	B, a, α	(46)	B, a, α	(70)	B, a, α	(43)
	Pre-sintered	1056	1049	528	504	550	548	527	520	547	529
		A, a, α	(53)	B, a, α	(83)	B, a, α	(50)	B, a, α	(51)	B, a, α	(75)
600 Grit SiC Paper grinded	Fully sintered	761	774	458	471	490	492	479	468	489	486
		A, a, β	(100)	B, a, β	(64)	B, a, α	(55)	B, a, α	(39)	B, a, α	(50)
	Pre-sintered	1050	1030	650	629	583	568	581	574	568	562
		A, a, α	(133)	B, a, α	(69)	B, a, α	(79)	B, a, α	(64)	B, a, α	(68)
Alumina particle air-abraded	Fully sintered	760	741	324	330	362	368	342	339	359	347
		A, a, β	(81)	B, b, α	(38)	B, b, α	(28)	B, b, α	(16)	B, b, α	(34)
	Pre-sintered	1128	1133	337	341	330	336	331	350	334	358
		A, a, α	(109)	B, b, α	(49)	B, b, α	(49)	B, b, α	(51)	B, b, α	(58)

**Table 5:** Medians, means and standard deviations in MPa of the biaxial flexural strength of test groups (N = 10) measured with ball-on-three-balls test. Statistically different medians (*p* ≤ 0.05) are indicated by different upper-case letters (within a column for analysis of the different materials with the same surface treatment), or by subscript lower-case letters (within a row for the same material with different surface treatment), or by different lower-case Greece letters (comparison of the cutting before and after sintering).

Treatment	Sintering Stage at Treatment	3Y-TZP		5Y-PSZ Enamel		5Y-PSZ TL1		5Y-PSZ TL2		5Y-PSZ Dentin	
		Median	Mean (SD)	Median	Mean (SD)	Median	Mean (SD)	Median	Mean (SD)	Median	Mean (SD)
1 µm polished	Fully sintered	841	860	574	570	546	550	578	564	537	514
		A, a, β	(131)	B, a, α	(43)	B, a, α	(49)	B, a, α	(43)	B, a, α	(99)
	presintered	1240	1226	662	607	595	584	618	616	662	654
		A, a, α	(78)	B, a, α	(83)	B, a, α	(60)	B, a, α	(50)	B, a, α	(53)
600 Grit SiC Paper grinded	Fully sintered	880	907	570	561	587	575	605	590	577	587
		A, a, β	(89)	B, a, α	(61)	B, a, α	(43)	B, a, α	(54)	B, a, β	(44)
	presintered	1362	1302	636	649	594	601	683	681	683	696
		A, a, α	(224)	B, a, α	(96)	B, a, α	(92)	B, a, α	(52)	B, a, α	(44)
Alumina particle air-abraded	Fully sintered	821	851	379	380	366	362	370	377	357	358
		A, a, β	(96)	B, b, α	(33)	B, b, α	(30)	B, b, α	(49)	B, a, β	(21)
	presintered	1350	1302	380	394	388	390	447	440	432	433
		A, a, α	(146)	B, b, α	(41)	B, b, α	(25)	B, b, α	(44)	B, b, α	(38)

## Data Availability

The data presented in this study are available on request from the corresponding author.

## References

[1] Manicone P.F., Rossi Iommetti P., Raffaelli L. (2007). An overview of zirconia ceramics: Basic properties and clinical applications. J. Dent..

[2] Denry I., Kelly J.R. (2008). State of the art of zirconia for dental applications. Dent. Mater..

[3] Pecho O.E., Ghinea R., Ionescu A.M., Cardona J.D.L.C., Paravina R.D., Pérez M.D.M. (2012). Color and translucency of zirconia ceramics, human dentine and bovine dentine. J. Dent..

[4] Kwon S.J., Lawson N.C., McLaren E.E., Nejat A.H., Burgess J.O. (2018). Comparison of the mechanical properties of translucent zirconia and lithium disilicate. J. Prosthet. Dent..

[5] Kosmač T., Oblak C., Jevnikar P., Funduk N., Marion L. (1999). The effect of surface grinding and sandblasting on flexural strength and reliability of Y-TZP zirconia ceramic. Dent. Mater..

[6] Monaco C., Tucci A., Esposito L., Scotti R. (2013). Microstructural changes produced by abrading Y-TZP in presintered and sintered conditions. J. Dent..

[7] Passos S.P., Linke B., Major P.W., Nychka J.A. (2015). The effect of air-abrasion and heat treatment on the fracture behavior of Y-TZP. Dent. Mater..

[8] Carrabba M., Keeling A.J., Aziz A., Vichi A., Fabian Fonzar R., Wood D., Ferrari M. (2017). Translucent zirconia in the ceramic scenario for monolithic restorations: A flexural strength and translucency comparison test. J. Dent..

[9] Aboushelib M.N., Wang H. (2010). Effect of surface treatment on flexural strength of zirconia bars. J. Prosthet. Dent..

[10] Michida S.M.D.A., Kimpara E.T., dos Santos C., Souza R.O.A., Bottino M.A., Özcan M. (2015). Effect of air-abrasion regimens and fine diamond bur grinding on flexural strength, weibull modulus and phase transformation of zirconium dioxide. J. Appl. Biomater. Func..

[11] Cheng M., Chen W., Sridhar K.R. (2003). Biaxial flexural strength distribution of thin ceramic substrates with surface defects. Int. J. Solids Struct..

[12] Ebeid K., Wille S., Hamdy A., Salah T., El-Etreby A., Kern M. (2014). Effect of changes in sintering parameters on monolithic translucent zirconia. Dent. Mater..

[13] Karakoca S., Yılmaz H. (2009). Influence of surface treatments on surface roughness, phase transformation, and biaxial flexural strength of Y-TZP ceramics. J. Biomed. Mater. Res. B.

[14] Börger A., Supancic P., Danzer R. (2002). The ball on three balls test for strength testing of brittle discs: Stress distribution in the disc. J. Europ. Ceram. Soc..

[15] Dewith G., Wagemans H.H.M. (1989). Ball-on-ring test revisited. J. Am. Ceram. Soc..

[16] Luthardt R.G., Holzhüter M., Sandkuhl O., Herold V., Schnapp J.D., Kuhlisch E., Walter M. (2002). Reliability and properties of ground Y-TZP-zirconia ceramics. J. Dent. Res..

[17] Wang H., Aboushelib M.N., Feilzer A.J. (2008). Strength influencing variables on CAD/CAM zirconia frameworks. Dent. Mater..

[18] Wille S., Zumstrull P., Kaidas V., Jessen L.K., Kern M. (2018). Low temperature degradation of single layers of multilayered zirconia in comparison to conventional unshaded zirconia: Phase transformation and flexural strength. J. Mech. Behav. Biomed..

[19] Grambow J., Wille S., Kern M. (2021). Impact of changes in sintering temperatures on characteristics of 4YSZ and 5YSZ. J. Mech. Behav. Biomed. Mater..

[20] Lumkemann N., Stawarczyk B. (2021). Impact of hydrothermal aging on the light transmittance and flexural strength of colored yttria-stabilized zirconia materials of different formulations. J. Prosthet. Dent..

[21] Chen B., Yan Y., Xie H., Meng H., Zhang H., Chen C. (2020). Effects of Tribochemical Silica Coating and Alumina-Particle Air Abrasion on 3Y-TZP and 5Y-TZP: Evaluation of Surface Hardness, Roughness, Bonding, and Phase Transformation. J. Adhes. Dent..

[22] Jerman E., Lumkemann N., Eichberger M., Zoller C., Nothelfer S., Kienle A., Stawarczyk B. (2021). Evaluation of translucency, Marten’s hardness, biaxial flexural strength and fracture toughness of 3Y-TZP, 4Y-TZP and 5Y-TZP materials. Dent. Mater..

[23] Huang C.W., Hsueh C.H. (2011). Piston-on-three-ball versus piston-on-ring in evaluating the biaxial strength of dental ceramics. Dent. Mater..

[24] Shin H.S., Lee J.S. (2021). Comparison of surface topography and roughness in different yttrium oxide compositions of dental zirconia after grinding and polishing. J. Adv. Prosthodont..

[25] Danzer R. (2014). On the relationship between ceramic strength and the requirements for mechanical design. J. Europ. Ceram. Soc..

[26] Amarante J.E.V., Pereira M.V.S., de Souza G.M., Pais Alves M.F.R., Simba B.G., dos Santos C. (2019). Roughness and its effects on flexural strength of dental yttria-stabilized zirconia ceramics. Mater. Sci. Eng. A.

[27] de Araújo-Júnior E.N.S., Bergamo E.T.P., Bastos T.M.C., Benalcázar Jalkh E.B., Lopes A.C.O., Monteiro K.N., Cesar P.F., Tognolo F.C., Migliati R., Tanaka R. (2022). Ultra-translucent zirconia processing and aging effect on microstructural, optical, and mechanical properties. Dent. Mater..

[28] Mao L., Kaizer M.R., Zhao M., Guo B., Song Y.F., Zhang Y. (2018). Graded Ultra-Translucent Zirconia (5Y-PSZ) for Strength and Functionalities. J. Dent. Res..

[29] Kolakarnprasert N., Kaizer M.R., Kim D.K., Zhang Y. (2019). New multi-layered zirconias: Composition, microstructure and translucency. Dent. Mater..

[30] Kontonasaki E., Giasimakopoulos P., Rigos A.E. (2020). Strength and aging resistance of monolithic zirconia: An update to current knowledge. Jpn. Dent. Sci. Rev..

[31] Inokoshi M., Vanmeensel K., Zhang F., De Munck J., Eliades G., Minakuchi S., Naert I., Van Meerbeek B., Vleugels J. (2015). Aging resistance of surface-treated dental zirconia. Dent. Mater..

[32] Börger A., Supancic P., Danzer R. (2004). The ball on three balls test for strength testing of brittle discs: Part II: Analysis of possible errors in the strength determination. J. Europ. Ceram. Soc..

[33] Schatz C., Strickstrock M., Roos M., Edelhoff D., Eichberger M., Zylla I.-M., Stawarczyk B. (2016). Influence of specimen preparation and test methods on the flexural strength results of monolithic zirconia materials. Materials.

